# A snapshot of how U.S. funding cuts impacted HIV care in one county of Kenya: a qualitative case study

**DOI:** 10.3389/fpubh.2026.1812962

**Published:** 2026-07-13

**Authors:** Erica Sedlander, Kyla Lawson, Njeri Wairimu, Kalin Werner, Sharon Mutai, Daniel Mwai, Monica Gandhi, Kenneth Ngure

**Affiliations:** 1 Institute for Health and Aging, University of California, San Francisco, CA, United States; 2 Independent Consultant, Sydney, NSW, Australia; 3 Kenya Medical Research Institute, Nairobi, Kenya; 4 Independent Consultant, New York, NY, United States; 5 Department of Medicine, University of California, San Francisco, CA, United States; 6 Jomo Kenyatta University of Agriculture and Technology, Nairobi, Kenya

**Keywords:** antiretroviral therapy, funding cuts, HIV, Kenya, United States

## Abstract

**Introduction:**

In January 2025, the United States government froze most foreign assistance and issued a 90-day stop-work order affecting many global health, education, and nutrition programs.

**Methods:**

Using a qualitative case study approach, we examined perceptions about experiences around how this abrupt funding disruption affected HIV care in one county in Kenya through in-depth interviews with women and men living with HIV and with healthcare providers.

**Results:**

Participants described immediate and severe antiretroviral therapy (ART) rationing and broader, cascading effects across the HIV care continuum, including missed visits, interrupted monitoring, and heightened anxiety about treatment continuity. Respondents also reported that rapid integration of HIV and primary care services—implemented amid the funding shock—undermined confidentiality in clinical settings, intensifying stigma concerns and discouraging care seeking among people living with HIV.

**Discussion:**

These findings highlight how sudden financing shocks can destabilize HIV programs in ways that extend beyond medication supply. We translate qualitative evidence into actionable recommendations to support person-centered HIV services during periods of uncertainty, including safeguarding continuous access to affordable/free ART, reducing access barriers such as transportation costs, and strengthening confidentiality protections as HIV and primary care services integrate.

**Systematic review registration:**

https://www.who.int/publications/i/item/9789241549684.

## Introduction

On January 20, 2025, the United States government froze all foreign aid and issued a 90-day stop-work order for virtually all global health, education, and nutrition projects (1, 2). Soon thereafter, the United States Agency for International Development (USAID), the largest organization implementing global health programs, was dissolved (3). USAID provided approximately 470 million dollars to Kenya annually, with about 80% of that budget going towards healthcare programs such as HIV, malaria, maternal health, and vaccinations (4, 5). On January 28th, the U.S. President's Emergency Plan for AIDS Relief (PEPFAR), the largest global HIV program, received a waiver allowing critical HIV treatment to resume; however, staff, infrastructure, and HIV drugs continued to be impacted (6). With this funding upheaval, the initial fear was the consequences of a disruption to antiretroviral drugs (ART) supply. ARTs suppress HIV, making it undetectable—and untransmittable (U = U) (7). Daily ART, a combination of HIV medications (usually administered in formulations of one pill a day), makes it possible to live a long, healthy life (8). Daily ARTs also allow women with HIV, including women in sero-different relationships (one partner living with HIV and the other not), to safely conceive and prevent vertical transmission to their babies during gestation and thereafter while breastfeeding (7, 9, 10). Without ARTs, HIV viral rebound occurs within days to weeks, and the disease can progress to AIDS (7). In more developed economies, such as the US, longer-acting ARTs like cabotegravir, rilpivirine, and lenacapavir, are available. However, in Kenya, where 3.3% of people live with HIV (11), and in the rest of sub-Saharan Africa, these newer agents are not widely available due to cost and lack of support from drug companies to distribute in low-income countries (12). Therefore, people living with HIV need to take a daily ART pill.

Several published anecdotal accounts reveal how individual people have been affected by the 2025 US funding cuts (13), including the estimated number of people impacted (14, 15). To our knowledge, this is the first qualitative study *to investigate how cuts have affected HIV care in Kenya*, joining two other qualitative studies on this topic from Malawi/South Africa and Uganda (16, 17). In this commentary, we report data from in-depth interviews with people living with HIV and the providers who care for them about how funding cuts affected HIV care in Thika, Kenya. This is an applied study aimed to inform program implementors and policy makers about the impact of the cuts and potential areas to focus future resources.

## Materials and methods

This sub-study was embedded in the larger parent study which was examining perceptions about how HIV and antiretroviral therapy (ART) affect fertility. Since the parent study's inception, US funding cuts occurred, so it was imperative to collect data on how these cuts affected HIV care prior to examining perceptions about ART and fertility. In-depth interviews took place in Thika, Kenya (population ~300,000) located about 45 kilometers from Nairobi. An estimated 2.4% of Thika residents are living with HIV (11, 18). Between August and November 2025, we conducted 20 in-depth interviews with men and women living with HIV and 10 with healthcare providers to understand how US funding cuts affected them and their communities. Men and women (ages 18–45 years) were purposively selected to balance gender and age distribution. Healthcare providers who care for men and women living with HIV were purposively selected to represent a range of: (1) healthcare facility types (public, faith-based, private, and research), (2) cadres and specializations (nurses, clinical officers, and doctors, including OB-GYN), and (3) service delivery points (prevention of mother-to-child transmission [PMTCT], maternal and child health [MCH], and comprehensive care clinic [CCC]). Men and women living with HIV were identified through ongoing and past research projects, nearby public healthcare facilities offering HIV services, and with support from a community health promoter (CHP). Study staff, in collaboration with healthcare providers and the CHP, approached potential participants, provided a brief description of the study, assessed interest in participation and conducted private verbal screening to determine eligibility. Eligible participants were aged 18–45 years, self-reported living with HIV, and expressed interest in future childbearing. Healthcare providers who care for men and women living with HIV were identified in collaboration with facility leadership from participating facilities and through existing research networks, including providers who had previously consented to future contact. Snowball sampling was used to recruit additional providers and ensure variation in clinical roles and facility types. A total of 28 participants were approached (12 men and 16 women); 20 were included in the study (10 men and 10 women). Non-participation was primarily due to availability and scheduling challenges.

For this sub-study, interview guides developed for people living with HIV explored awareness of US support for HIV services, perceptions of recent funding changes, and impacts on care. Providers were asked how funding cuts affected their work and their hopes and concerns for HIV and healthcare more broadly. Researchers from the US and Kenya, developed the interview guides together. Guides were pilot tested and revisions were made following the pilot interviews (see Supplementary Appendix 1).

### Data collection

Between August and November 2025, interviews were conducted in-person at the Kenya Medical Research Institute (KEMRI) offices, in English with providers, and in Swahili with people living with HIV. All interviews were audio recorded, transcribed, and translated into English. Trained, gender-matched interviewers (NW, DM, SM) with extensive qualitative experience, who live in Kenya, conducted the interviews, which each lasted approximately 1 h. Participants received compensation equivalent to $4 USD for men and women living with HIV and $8 USD for healthcare providers.

### Data management and analysis

Interviewers completed post-interview reports to document emerging themes. Data collection and analysis happened concurrently to ensure that saturation was reached. Specifically, we began listening to interviews and reading transcripts as soon as they were transcribed by the coding team (KL, KW, ES). We used thematic analysis (19) to identify emerging themes through an iterative approach that combined data collection and analysis concurrently to ensure thematic saturation (20). Both inductive and deductive coding were applied with a priori codes related to funding cuts such as “system level changes due to funding cuts” and “impact to HIV treatment” supplemented by emergent themes such as integration of HIV and primary care, how funding cuts exacerbated HIV stigma, and working within resource constrained settings. The original, pre-determined codes (deductive coding) align closely with the interview guide while the emerging codes (inductive coding) arose during coding.Two researchers independently coded the first interview starting with a priori codes but added emerging codes as well and discussed changes to the codebook during regular meetings with the whole analysis team (21). Analytic memos were written after each coded interview to capture insights and emerging themes. Memos, post-interview reports, and code reports were triangulated to develop final themes. In all analysis methods, we compared responses from healthcare providers and men and women living with HIV to uncover similarities and differences.

### Ethical considerations

Ethical approval was obtained from Institutional Review Boards at the University of California, San Francisco (#23-38343) and Kenya Medical Research Institute (KEMRI/SERU/CCR/0386/5128). Prior to any data collection, written informed consent was obtained from all participants.

## Results

## Participant characteristics

The sample was evenly split between men and women with a younger mean age for men (23 years) compared to women (28 years). Most participants were married or in long-term relationships. The majority completed secondary school or completed some college. Two-thirds of the sample had children and one third did not. All participants were currently taking ARTs but almost half had missed ART doses at some point. See Table 1 for a full description of the sample.

**Table 1:** Demographics of men and women living with HIV.

	Total	Male	Female
	*n* (%)	*n* (%)	*n* (%)
Total responses	20 (100)	10 (100)	10 (100)
Gender			
Women	10 (50)	–	10 (100)
Men	10 (50)	10 (100)	–
Age			
Mean (range)		22.5 (22–44)	28.2 (19–44)
Marital status			
Single (never married)	7 (35)	4 (40)	3(30)
Single (in a relationship)	2(10)	–	2(20)
Married	11 (55)	6 (60)	5 (50)
Level of education			
Some primary school	1 (5)	1 (10)	–
Completed primary school	2 (10)	1 (10)	1 (10)
Some secondary school	1 (5)	–	1 (10)
Completed secondary school	8 (40)	4 (40)	4 (40)
Some or completed tertiary/college education	8 (40)	4 (40)	4 (40)
Employment status			
Formal employment	3 (15)	3 (30)	–
Casual jobs	5 (25)	2 (20)	3 (30)
Self-employed/business	8 (40)	4 (40)	4 (40)
Unemployed	4 (20)	1 (10)	3 (30)
Religion			
Christian	18 (90)	8 (80)	10 (100)
Muslim	2 (10)	2 (20)	–
Has children			
No	7 (35)	3 (30)	4 (40)
Yes	13 (65)	7 (70)	6 (60)
Number of children			
Average (range)		1.9 (0–4)	1.1 (0–4)
Currently taking ARTs			
No	–	–	–
Yes	20 (100)	10 (100)	10 (100)
Ever missed ART doses			
Never	12 (60)	7 (70)	5 (50)
Once	1 (5)	–	1 (10)
A few times (3 or less)	4 (20)	1 (10)	3 (30)
Several times (missed 5–10)	2 (10)	1 (10)	1 (10)
Many times (missed more than 10)	1 (10)	1 (10)	–

Healthcare providers were almost evenly split by gender. Most providers worked in public or faith-based hospitals. Providers represented a mix of nursing, clinical officer, and physician roles. Providers had extensive experience delivering healthcare, with a mean of nearly 16 years in practice. See Table 2 for a full description of the sample.

**Table 2:** Demographics of healthcare providers in our study sample.

	*n* (%)
Gender	
Women	6 (60)
Men	4 (40)
Health care setting	
Public hospital	6 (60)
Faith-based hospital	2 (20)
Research institution	1 (10)
Private clinic	1 (10)
Training	
Nurse	4 (40)
Clinical medical officer	2 (20)
Medical doctor/OB GYN	3 (30)
Health coordinator	1 (10)
Years of providing healthcare	
Mean (range)	15.7 (5–37)

Participants described a range of changes to HIV care due to funding cuts which we categorized into four themes.

## Rationing ART supply resulted in more frequent visits to the pharmacy affecting myriad health and quality of life factors

Given that the funding freeze occurred without notice, ART supply systems were unprepared to maintain pre-freeze levels. Most participants, including men and women living with HIV and medical providers, reported ART rationing, with refill intervals reduced from 6-month supplies to monthly, or in some cases, for just 1 to 2 weeks at a time.

A man living with HIV's lived experience included the following

“*So, it [funding cuts] affected me, because I was getting medication for 3 months, and when that happened, it dropped from 3 months to 7 or 14 days”* (37-year-old man).

This change was more disruptive than one might imagine. Another man described that increased clinic visits led to unemployment when his employer refused repeated time off.

“*I was told that I would only get them (ARTs) every month. At that time, I was employed and I could not come here every month. So, I had to ask for permission so asking for permission for like two months, it cost me my job. I lost it”* (37-yearold man).

Ultimately, he was forced to choose between employment and accessing lifesaving medication.

Frequent clinic visits posed substantial financial and logistical barriers, particularly for those living far from health facilities. Participants, especially women, reported that transportation costs forced delayed visits and missed doses. One woman stated,

“*People used to be given [public transportation] fare. Now that there is no funding, most of the people who are failing to come, it's because of* the *fare. So it [funding cuts] contributed a lot, you find someone's viral load has risen, they missed drugs, or they have started getting sick again*” (20-year-old woman).

Another woman reported that her clinic ran out of ARTs and infant prophylaxis, forcing her to stop breastfeeding despite prior counseling that breastfeeding was safe with consistent treatment (10). The woman said,

“*When I got to the hospital, I wastold there is no medicine. I asked, “So what will I do?” They told me, “Now you will have to stop the child from breastfeeding.” That's how I stopped my child from breastfeeding when he was seven months old”* (23-year-old woman).

Healthcare providers also described being forced to switch from patients' original regimens to non-standard regimens because of drug unavailability, resulting in new or worsening side-effects. One provider elaborated on challenges posed stating,

“*Atazanavir is a second line... a PI (Protease Inhibitor) based regimen but during that crisis we were forced to switch patients because we could not access Atazanavir. We could source from all other facilities, but we were not able to get them so sometimes we are forced to change them to non-standard*” (Clinical Officer).

Patients corroborated these experiences of rapidly altering medications,

“*There is a time when these drugs [ARTs] were not available, so at the end of the day, you get drugs, but it's not fair to get this drug today, tomorrow I have another one, because they give different side effects. There are some you will feel itchy, some will giveyou headache, some will make you feel like vomiting*” (33-year-old woman).

### More frequent health center visits affected staff workload

More frequent refill schedules increased provider workload and staffing strain. As one provider explains further,

“*We've had a lot of people trying to get different facilities, maybe to register after the US government-funded facilities were closed. Having only two weeks supply means that we get a lot of our clients coming over and over again, it's a workload issue*” (Study clinician).

## Shutting down HIV-specialty centers and integration with primary care impacted confidentiality

Following USAID cuts, many people living with HIV were shifted from HIV-specific comprehensive care clinics (CCC) to general outpatient pharmacies. Participants reported that this increased the risk of inadvertent disclosure of HIV status, particularly in community pharmacies, exacerbating stigma and distress. Respondents spoke about the lack of knowledge around HIV and stigma in their larger community. As a result, many limited disclosure of their HIV status to close family members and found public medication collection threatening to their privacy. Although these changes were not intended to compromise confidentiality, participants consistently identified this as a barrier to care, such as getting their ARTs in a “*brown bag*,” at the pharmacy, or

“*meeting your neighbor in the same queue and the medication that you are going to be given, automatically it [has ART] is written on it* (36-year-old man).”

One man living with HIV went on to elaborate on the reality of this,

“*It [integration] is going to affect many people because there is still that anxiety of people interacting with those who are HIV positive. And you know that psychological torture of people who are not positive and the way they look at you, as if there is a mistake that we made. If you do business, for example, people will avoid you because of that HIV status*” (36-year-old man).

Providers corroborated these sentiments about integration affecting privacy and care. As one provider highlights, occasions of potential stigma are influencing medication decision making,

“*We're being told to integrate those services, and you know they used to be given their ARTs from the CCC, now they have to go to the pharmacy and it's a really really big challenge to them because there are issues with stigma, so they feel like it's no longer private to them. Some of them might even go without taking their drugs*” (Nurse).

Providers also mentioned the need to pivot roles quickly and the pressures of integration because

“*some staff lost their jobs*” (Medical doctor).

The workload when teams are short staffed is often difficult to navigate; this coexists with fears of further job cuts, and no prospect of hiring to full capacity.

## Funding cuts engendered fears about the future of HIV and calls for the Kenyan government to cover the deficit

Men and women living with HIV feared ART inaccessibility due to stockouts and unaffordable out-of-pocket costs. Some participants mentioned that paying for the medications themselves felt like a potential death sentence and they would be left “without any hope.” One man living with HIV said,

“*Now you don't know if they will be paying, and some of us we cannot afford those medications. And then there are also those emotions; what will it happen now? What is the next step because this is our life, those medications are our lives, if we stop taking them or we have to pay, then I think that a lot of people are going to die*” (36-year-old man).

Another woman spoke further of psychologial benefits ART provides her knowing she is not capable of transmitting the virus,

“*If not for this drug, I cannot live. Because this is like my second God. And without that drug, I will infect many people. If I infect one, I don't know if they are honest and they will go and infect another*” (23-year-old woman).

Providers feared losing “all progress” in fighting the HIV epidemic. Staffing losses would undermine HIV control by reducing care and testing capacity, leading to increased infections and lower virologic suppression rates.

One provider shares their future concerns,

“*We had it [HIV] under control. My fear is we'll have more infections, premature deaths, mother-to-child transmission so that will be a crisis for us. And now for us healthcare workers in case you happen to get a prick...needle during surgery, there is no fallback mechanism, no PEP [post-exposure prophylaxis] for you, so that might be crazy for us*” (OB-GYN).

### Desire for the Kenyan government to cover the deficit

Both people living with HIV and healthcare providers, expressed a desire for the Kenyan government to assume greater responsibility for HIV financing and service delivery,

For example, one man stated,

“*If the government can, these research hospitals, they can come up with a full solution, it is not a must to depend on outside countries [foreign aid]. If the government can come out and help researchers, they can come up with ideas that can help us locally*” (37-year-old man).

A provider agreed with this sentiment,

“*As a country, we should be thinking of manufacturing our own ART. We should stop this overdependence; it cripples us as a nation. Yes, it may be costly in the beginning, but it won't be as expensive as it will be when those people suddenly withdraw the way they did in January*” (Medical doctor).

Collective opinions priortitised the need to reduce dependence on foreign aid, despite skepticism about its current capacity to do so.

## Stockouts and higher costs of condoms meant people could not use barrier methods to prevent pregnancy and sexually transmitted infections like HIV

Funding cuts also affected access to condoms and other contraceptives, which were previously provided at no cost. Rising out-of-pocket costs drove use of lower-cost nonpreferred options (e.g., condoms and oral contraceptive pills), which were still sometimes unavailable. Due to the cost, previously preferred longer acting methods like injections, were financially out of reach. Condom shortages and higher costs increase HIV transmission and unintended pregnancy (22). One man indicated the personal cost of safer sexual practices,

“*And still there have been no condoms since January, so it became an expense to us as we had to go back to our pockets in this economy. So, it affects us a lot* (37 year-old man).”

A provider corroborated this,

“*There are no condoms. Things like family planning is not available* (Nurse).”

## Discussion

Our findings highlight the cascading effects of US funding cuts on people living with HIV and the providers who care for them in Kenya. While disruptions to ART supply were anticipated, participants described unanticipated consequences including more frequent pharmacy visits resulting in job loss and breaches of confidentiality following rapid integration of HIV and primary care services. This snapshot from Kenya provides timely insights for policymakers and program planners navigating future funding transitions.

Our findings align with prior qualitative research from South Africa and Malawi documenting service-level disruptions across HIV services, including testing, treatment, retention, and support (16). These countries had documented suspensions of HIV prevention commodities (e.g., condoms) and shifts in HIV care from specialized clinics to public facilities, but largely focused on logistical challenges rather than downstream social and economic effects. Our findings extend this literature by capturing how service disruptions affected patients' livelihoods and well-being. Another qualitative study in Uganda similarly found that closing facilities, in their case community based pharmacies where people could go at night, meant that people with HIV who feared that others would know their statues, likely would not go to facility-based care (17). Additionally, a youth-led quantitative survey across 18 Kenyan counties similarly reported widespread service disruptions following the funding freeze, with 86% noting reduced access to HIV services, shortened ART refill intervals, increased transportation costs, and treatment fatigue (23). Our analysis builds on this work by incorporating perspectives of Kenyan healthcare providers and providing qualitative data around how and why the funding cuts impacted them.

In September 2025, while some of these interviews were being conducted, the US released a new America First Global Strategy, prioritizing foreign aid aligned with US interests, including infectious disease control and pharmaceutical exports (24). HIV prevention aligns with this approach as transmission is transnational (25). The strategy also emphasizes partner-country self-reliance, echoing participant perspectives and similar findings in the recent literature (24, 26, 27). However, abrupt funding withdrawal without transition planning has ramifications. On December 4, 2025, the US Department of State and Kenya signed a $2.5 billion, 5-year health operation framework to support this transition. Kenya is the first African country to successfully negotiate a new health partnership with the United States (28). The Government of Kenya committed an additional $850 million in domestic health spending over the course of the 5-year framework.

Our findings contribute to the Health Stigma and Discrimination Framework [Stangl et al. (29)] in two ways. First, they suggest that funding shocks can be an upstream driver of anticipated stigma (29). We found that the rapid integration of HIV and primary care without proper consideration of person-centered confidential care, resulted in anticipated stigma. People living with HIV feared that they would not be able to maintain their confidenitlity outside of HIV specific facilitites. These findings add specificity and empirical evidence around financing shocks as upstream structural drivers of anticipated stigma to the broader framework. Second, our data illustrates that the cascade of disruption led to rationing ART, which resulted in more visits to health centers, thereby missing work and additional transportation barriers, which translated to missed ART doses potentially affecting viral rebound and transmission. This cascade effect caused specifically by US funding cuts has not been empirically shown representing clear program and policy implications.

Our findings which include insights from multiple stakeholders (men and women living with HIV and provders from myriad specialties and health centers) point to specific recommendations as local and national governments decide where to intervene during this transition period (30). First, although integration of HIV services into primary care may be cost-effective (26, 31), implementation must be sensitive to the needs of people living with HIV. Because the integration of HIV and primary care accelerated with US funding cuts, the rollout, from the perspective of participants in this study, was not person-centered. Specifically, confidentiality did not appear to be a priority despite prior research showing that stigma negatively affects care seeking (29). Patient centered, stigma-reducing care could look like generic packaging for all medications including ARTs. Secondly, given that medical providers who are not accustomed to interacting with HIV patients may be caring for them, perhaps training for all healthcare providers on stigma-reducing care is warranted. Third, developing participant advisory groups that include both healthcare providers and people living with HIV to provide feedback on health integration and independence implementation could ensure that care is person-centered. Finally, in addition to condoms, ensuring that ARTs are free and well stocked to obviate excessive trips to the health center and payment for transportation fare to get to the health center for those who cannot afford it is essential. More stockpile of lifesaving medications (including ART and condoms) and a contingency plan if additional funding shocks occur, could also prevent some of the negative effects on HIV care and its sequlea.

This study has several limitations. Data were collected in a single county of Kenya and did not include policymakers, limiting generalizability and policy insights. Nonetheless, our interviews conducted within 6 months of the funding cuts provide a timely, in-depth perspective from affected patients and providers. This study illustrates where resources could be allocated (e.g., support for ART access, including availability, cost, and transportation, infrastructure that respects confidentiality, and increased staff time as HIV and primary care integration unfolds). As the Kenyan and US governments embark on the new 5-year plan to transition to independence, our findings can inform strategies to ensure that plans consider the unique needs of people living with HIV and the providers who care for them.

## Data Availability

The raw data supporting the conclusions of this article will be made available by the authors, without undue reservation.
