# Supplementary material for: A snapshot of how U.S. funding cuts impacted HIV care in one county of Kenya: a qualitative case study
Source: Front Public Health. 2026 Jul 13;14:1812962. doi: 10.3389/fpubh.2026.1812962 (PMC13402901; doi:10.3389/fpubh.2026.1812962)
Supplement: Supplementary file 1 [file Table_1.docx]

Thank you again for being here today. I want to ask you about recent changes in HIV and reproductive health care and how it affects your work.

**Section 1. Recent changes in HIV and fertility care in Kenya**

- Since January 2025, have you noticed any changes to your work? Probe: this could be changes to your facility, availability of resources, or your work specifically. Probe: What have you heard about changes in funding related to HIV and reproductive health?
- Can you tell me about how these changes have affected *HIV treatment efforts? (e.g., ART)*
- Can you tell me more about how these changes have affected *reproductive health services* (e.g. contraception, diagnostic testing for infertility, and infertility treatment)?
- Looking ahead, what are your main concerns for HIV and reproductive work in Kenya given recent changes?

## Now I’m going to start by talking about getting pregnant and having children. I’ll ask you some questions about what your patients believe (this could be different from what you believe).

## Section 2: Social norms around childbearing and infertility

- In general, among your patient population, what are the expectations for men and women to have children? Probes: How many? When?
- How much of a problem is infertility among your patients?
- Can you tell me about how you speak to your patients about their infertility?
- If a couple is infertile in this community, what are all the possibilities that can happen to the woman? Probe: What usually happens to the *woman*?
- If a couple is infertile in this community, what are all the possibilities that can happen to the man? Probe: What usually happens to the *man*?
- How are men and women affected differently by infertility in this community?

**Section 3: Perceptions about causes of infertility more generally (e.g., untreated sexually transmitted infections, advanced age, endometriosis**

- What do your patients think is a normal time range to get pregnant? When is it considered too long (how many months or years)?
  - Follow-up question: When does a couple start worrying about becoming pregnant?

Now I’m going to ask about **YOUR** beliefs, not your patients. Reminder that we are just trying to understand your general thoughts. This is not a test. ☺

- How would you *define* infertility?
- What do *you* think *causes* infertility?
- Thank you. The definition of infertility is not getting pregnant after a year of having sex without family planning.
- Infertility is often caused by untreated STIs like chlamydia and gonorrhea, pelvic inflammatory disease (PID). Other causes include fibroids, advanced age, endometriosis, postpartum or post-abortion infection, invasive gynecological procedures, and some cases have an unknown cause.

**Section 4: Knowledge and perceptions about safe conception and vertical transmission.**

- Can *you* tell me your thoughts about how women living with HIV can safely conceive and give birth without passing it to their partner or baby? Probe: if they have not mentioned ART: what do women have to do to make sure their partner (if they are sero-discordant) or baby does not get HIV?
- What do your patients know about how women living with HIV can safely conceive and give birth without passing it to their partner or baby?
- What do your patients friends, family, or community know about this? Probe: Besides your patients, what do other people living with HIV think about this?

**Now I’m going to ask questions about men and women living with HIV specifically and their fertility.**

**Section 5: Knowledge and perceptions about the biological capacity of men and women living with HIVs ability to conceive and how ART affects that relationship.**

First, I’ll ask you about ***your*** thoughts then move onto your patients.

- From your perspective, how does HIV affect a woman’s ability to get pregnant?
  - Probe: do women with HIV have higher rates of infertility?
- How does taking ART impact a woman’s chances of getting pregnant?
- From your perspective, how does HIV affect a man’s ability to get a woman pregnant?
  - Probe: do men with HIV have higher rates of infertility?
- How does taking ART impact a man’s chances of getting his partner pregnant?

Now I’ll ask you about your patients.

- What do *your patients* know about how HIV impacts their ability to get pregnant?
  - Probe for knowledge among men and women living with HIV
- What are *their perceptions* about how taking ART impacts their chances of getting pregnant? Are you aware of any negative beliefs about ART and fertility? Probe: can you tell me about them?
  - Probe for perceptions of both men and women living with HIV

## Section 6: How providers discuss fertility with their patients

- How do you discuss *fertility* with your patients living with HIV? Probe: Tell me about how you discuss fertility with your female patients? Probe: Tell me about how you discuss fertility with your male patients? Probe: Does it differ for men and women?
- Can you tell me how you discuss the impact of taking ART on your patients’ chances of getting pregnant? Probe: How do those conversations play out? Probe: typically, what are the questions that your patients ask? Probe: How do these conversations differ for men and women?

Thank you. Men and women living with HIV have higher rates of infertility compared to people without HIV. This can be due to prolonged anovulation and amenorrhea, and increased risk and severity of other co-infections, such as chlamydia and gonorrhea, which can lead to pelvic inflammatory disease (PID), a leading cause of tubal factor infertility. HIV also affects sperm quality. Fortunately, ART (for both men & women), improves a woman’s chances of getting pregnant.

Now I’m going to ask your recommendations about how to educate men and women about how HIV affects their fertility.

**Section 7. Recommendations for sensitization around HIV, ART and fertility**

- To start with, what is the best way to raise awareness about the general causes of infertility outside of HIV? For example, untreated sexually transmitted infections, PID, endometriosis, fibroids, advanced age, post-partum or post abortion scarring, etc.
- Our hypothesis is that if women knew that HIV negatively impacted their chances of getting pregnant, but ART improves their chances, they will be more likely to adhere to ART. What is a good way to educate men and women living with HIV about how it impacts their fertility? Why that way? Probe: what is the best way to reach women specifically? How about men?
- Who is the best person to share this message (e.g., a medical doctor, a clinical officer, community health worker, peer, etc.? Why that person?
- Would this be something that a provider like yourself could communicate? Why would that be easy or hard to do?
- What should be included in this messaging? What would help you or your patients deliver information about fertility and HIV?

That’s the end of the interview. Do you have anything else you would like to add?

Thank you for your time.
