# Supplementary material for: A snapshot of how U.S. funding cuts impacted HIV care in one county of Kenya: a qualitative case study
Source: Front Public Health. 2026 Jul 13;14:1812962. doi: 10.3389/fpubh.2026.1812962 (PMC13402901; doi:10.3389/fpubh.2026.1812962)
Supplement: Supplementary file 2 [file Table_2.docx]

Thank you again for taking the time to be here today. I want to ask you about changes in HIV and reproductive health care in Kenya. I’ll ask you about any changes you have experienced as part of your HIV care.

**Section 1. Recent changes in HIV and fertility care in Kenya**

- Since January 2025, have you noticed any changes to the health care you receive? Probe: this could be HIV or reproductive health care. Probe: What have you heard about changes in funding related to HIV and reproductive health?
- Can you tell us about how these changes have impacted people living with HIV’s ability to get HIV care (e.g. ART)? Probe: has anyone you know run out of ART or received fewer bottles of ART at a time?
- Looking to the future, what are your hopes or concerns for receiving HIV and reproductive health care in Kenya?

We are now going to talk about getting pregnant and having children. I’ll ask you some questions about what people in the community where you live believe (this could be different from what you believe).

## Section 2: Social norms around childbearing and infertility

- In general, how many children are men and women expected to have in the community where you live? When do people in your community think it is the right or best time to have kids? Probe: at what age? How soon after marriage?
- What do you know about people in this community having trouble (issues) getting pregnant? Probe: Is having trouble (issues) getting pregnant common in your community?

## Vignettes

## Now I’m going to tell you a story about a couple in a community like yours who want to have a baby. I would like you to share your thoughts on the situation.

Caro is 28 years old and married to Bryan who is 30 years old. They have been trying to get pregnant since they got married three years ago but have not been able to. After three years, they are worried.

- Who would they turn to for information or help? Probe: What would the woman do? Probe: What would the husband do?
- What would happen to their relationship?
- What kinds of things would people in their community say to the woman?
- What kinds of things would people in their community say to the man?
- How does the community react differently to the man versus the woman having trouble getting pregnant?

Now I’ll ask your personal opinions about pregnancy and having children NOT your community’s opinions.

**Section 3: Perceptions about causes of infertility more generally (e.g., untreated sexually transmitted infections, advanced age, endometriosis**

- When a couple is trying to get pregnant, what do ***you*** think is a normal time range to get pregnant?
  - Probe: When is it considered too long (how many months or years)?
  - Probe: When does a couple start worrying about becoming pregnant?
- What can make it difficult for someone to get pregnant in general? In other words, why does it take a long time for couples to get pregnant? Or why can’t some couples get pregnant at all?
- Thank you. The definition of infertility is not getting pregnant after a year of having sex without family planning. Infertility is often caused by untreated STIs like chlamydia, gonorrhea which can lead to pelvic inflammatory disease (PID). Other causes include fibroids, advanced age, postpartum or post-abortion infection.

**Now I’m going to ask some questions about men and women living with HIV and getting pregnant.**

**Section 4: Knowledge and perceptions about safe conception and vertical transmission.**

- Can you tell me how women living with HIV can safely get pregnant and give birth without passing it to their partner or baby? Probe: if they have not mentioned ART: what do women have to do to make sure their partner (if they are sero-different) or baby does not get HIV?
- What do people in your community know about this? What do your friends and family know about this? How about others living with HIV?

**Section 5: Knowledge and perceptions about the biological capacity of women living with HIVs ability to conceive and how ART affects that relationship.**

- In your opinion, how does HIV affect a woman’s ability to get pregnant? Probe: do women living with HIV have a harder time getting pregnant?
- In your opinion, how does HIV affect a man's ability to get a woman pregnant? Probe: in other words, can you tell me about whether men living with HIV have a harder time getting their partner pregnant than men without HIV?
- How does HIV treatment (antiretroviral therapy (ART) affect a woman living with HIVs chances of getting pregnant?
- What about men living with HIV? Probe: How does HIV treatment (antiretroviral therapy (ART) affect a man living with HIVs chances of getting his partner pregnant?
- What do *your friends and family* know about how HIV affects women’s ability to get pregnant? Probe: What do they know about how HIV affects a men’s ability to get a woman pregnant?

Now I’m going to talk to you about how your doctor, nurse, or community health worker talks to you about HIV and your or your partners ability to get pregnant.

## Section 6: How providers discuss fertility with their patients

- How has your doctor, nurse, or community health worker discussed *pregnancy or having kids* with you since you have been diagnosed with HIV? Probe: Since you’ve been diagnosed with HIV, how has it come up in your appointments? Probe: what information were you given?
- How has your doctor, nurse, or community health worker talked about how HIV affects your chances of getting pregnant/getting a woman pregnant? Probe: How do those conversations play out?
- If yes, how did you feel after that conversation?
- If yes or no, how would you like that discussion to go?

I want to tell you that men and women living with HIV have a harder time getting pregnant than people without HIV. BUT if they take their HIV medications (antiretroviral therapy – ART) regularly, their chances of getting pregnant are about the same as people without HIV. In other words, if people with HIV want to get pregnant and have kids, then taking ART is important.

Now I’m going to ask your recommendations about how to educate men and women with HIV about how HIV affects your or your partners ability to get pregnant.

**Section 7. Recommendations for sensitization around HIV, ART and fertility**

- What is a good way to educate men and women living with HIV that if they want to get pregnant any time in the future, taking ART as soon as they are diagnosed is important? Probe: Why that way?
- Who is the best person to share this message (e.g., a medical doctor, community health worker, peer, etc.)? Probe: Why that person?
- As mentioned before, aside from HIV, other things can cause infertility like untreated sexually transmitted infections (STIs) at any point in someone’s lifetime, older age, and postpartum or post-abortion infections. Both men and women contribute to infertility. There are often misconceptions about what causes infertility. What is the best way to raise awareness among men and women like you that all these other things can make it difficult to get pregnant?

Is there anything that we have not talked about that you would like to share?

Thank you for taking the time.
